# Age-dependent alpha-synuclein accumulation and aggregation in the colon of a transgenic mouse model of Parkinson’s disease

**DOI:** 10.1186/s40035-018-0118-8

**Published:** 2018-06-30

**Authors:** Qian-Qian Chen, Caroline Haikal, Wen Li, Ming-Tao Li, Zhan-You Wang, Jia-Yi Li

**Affiliations:** 1 Institute of Neuroscience, College of Life and Health Sciences, Northeastern University, 110819 Shenyang, People’s Republic of China; 2 Neural Plasticity and Repair Unit, Wallenberg Neuroscience Center, Department of Experimental Medical Science, Lund University, BMC A10, 22184 Lund, Sweden; 3 Guangdong Province Key Laboratory of Brain Function and Disease, Zhongshan School of Medicine, Sun Yat-sen University, No. 74 Zhongshan Rd.2, Guangzhou, 510080 China; 4 Institute of Heath Sciences, China Medical University, 110112 Shenyang, People’s Republic of China

**Keywords:** Parkinson’s disease, Colon, α-syn, Phosphorylation, VIP, nNOS, Calretinin, Enteric nervous system

## Abstract

**Background:**

Parkinson’s disease (PD) is one of the most common neurodegenerative diseases, neuropathologically characterized by misfolded protein aggregation, called Lewy bodies and Lewy neurites. PD is a slow-progressive disease with colonic dysfunction appearing in the prodromal stage and lasting throughout the course of the disease.

**Methods:**

In order to study PD pathology in the colon, we examined the age-dependent morphological and pathological changes in the colon of a PD mouse model expressing human wildtype α-synuclein (α-syn) fused with the green fluorescent protein (GFP), under the endogenous mouse α-syn promoter.

**Results:**

We observed an age-dependent progressive expression and accumulation of α-syn-GFP in the enteric neurons of Meissner’s (submucosal) and Auerbach’s (myenteric) plexuses of the colon. Additionally, the phosphorylation of α-syn at serine 129 also increased with age and the aggregation of α-syn-GFP coincided with the appearance of motor deficits at 9 months of age. Furthermore, α-syn (-GFP) distinctly co-localized with different subtypes of neurons, as identified by immunohistochemical labeling of vasoactive intestinal peptide (VIP), neuronal nitric oxide synthase (nNOS), and calretinin.

**Conclusions:**

Our results show the development of α-syn pathology in the enteric neurons of the colon in a PD mouse model, which coincide with the appearance of motor deficits. Our mouse model possesses the potential and uniqueness for studying PD gastrointestinal dysfunction.

**Electronic supplementary material:**

The online version of this article (10.1186/s40035-018-0118-8) contains supplementary material, which is available to authorized users.

## Background

Parkinson’s disease (PD) is a common neurodegenerative disease of ageing, pathologically characterized by the loss of dopaminergic neurons in the substantia nigra pars compacta [1] and the formation of intracellular inclusions termed Lewy bodies (LBs) and Lewy neurites (LNs) [2, 3]. Immunohistochemical analyses have shown that misfolded α-syn is the primary protein component of LBs and LNs [4], which suggests its potential role in the pathogenesis of PD. Typical motor symptoms of PD include tremor, rigidity, bradykinesia, and postural instability. Most, if not all, PD patients also suffer from multiple non-motor features such as depression, rapid eye movement sleep behavioral disorder, excessive daytime sleepiness, constipation, etc [5]

Recent evidence suggests that PD pathology can spread from the gut to the brain [6]. Braak and colleagues first proposed this hypothesis based on their systemic examinations of tissues from the peripheral organs to the brain: Lewy pathology in the intestinal tract and lower brain stem preceded the appearance of pathology in the upper brain regions in postmortem tissues of PD patients [7, 8]. Recently, we showed direct evidence of PD pathology spread from the gastrointestinal tract to the brain in a rat model after exogenous α-syn was injected into the intestinal wall [6]. In addition, retrospective statistical analyses showed that full truncal vagotomy diminished the risk of subsequent PD development, suggesting that the vagal nerve, the neuronal connection between the gut and brain, is a route for PD pathology spread [9]. Moreover, our previous study demonstrated that α-syn was more highly expressed in the distal segment of the small intestine than in the proximal segment. It is consistent with the distribution of the microbiota that: the stomach and upper small intestine are essentially sterile; the bacteria count rises further along the intestine [10]. Considering that the large intestine contains the largest bacterial ecosystem in the human body [11], it inspired us to further explore whether pathological alteration of α-syn is present in the enteric nervous system of the colon, which plays an essential role in the development of constipation in early PD.

## Materials and Methods

### Mouse strain

The transgenic mouse model used in this study has been described previously [12, 13]. Briefly, BAC-α-syn-GFP-transgenic mice were generated by pronuclear injection of BAC DNA into fertilized eggs of C57 black 6 (C57BL/6) mice. The BAC DNA is composed of the human full-length wildtype α-syn gene fused with the GFP sequence. The human α-syn cDNA was inserted into the pAcGFP1-C1 vector at the initiation codon of the mouse α-syn gene.

### Tissue preparation

BAC mice aged 3 to 24 months (3 m, *n* = 3; 6 m, n = 3; 9 m, n = 3; 18 m, *n* = 1; 24 m, n = 3) and C57BL/6 wildtype mice (6 m, n = 3; 9 m, n = 3; 18 m, n = 3) were sacrificed by transcardial perfusion with 0.1 M phosphate buffer solution (PBS), followed by 4% paraformaldehyde (PFA). The colon was dissected and post-fixed in 4% PFA overnight. Fixed colon was then transferred to 10, 20, 30% sucrose in 0.1 M PBS, consecutively. The colon was then cut transversely into 10 μm sections using a cryostat (Leica, Germany) and mounted on gelatin-coated glass slides.

### Immunohistochemistry and analyses

Antigen retrieval of the samples was performed in heated citrate buffer (pH 6). Endogenous peroxidase was blocked by treatment with 3% hydrogen peroxide and 10% methanol in 0.1 M PBS at room temperature for 15 min. The sections were then pre-incubated with blocking solution (5% normal goat serum and 0.3% Triton X 100 in 0.1 M PBS) for 1 h at room temperature. Primary antibody (anti-α-syn (phosphoS129), Abcam, ab51253, rabbit, dilution: 1/500), was incubated on samples at 4 °C overnight, followed by incubation with the secondary antibody (biotinylated goat anti-rabbit IgG antibody, Vector Laboratories, BA-1000, dilution: 1/500) at room temperature for 1 h, and ABC solution (Vector Bio labs, Cat. # VEPK-6100) at room temperature for 1 h. The staining was developed with diaminobenzidine (VMR chemical, Cat. # 0430, 0.03% dissolved in 0.05 M pH 7.5 tris-HCl) for 5 min. The sections were then dehydrated sequentially with 70, 80, 90, 95,100% ethanol and dimethyl benzene, then mounted with neutral balsam for microscopic analyses. In each round of immunohistochemical staining, we included “negative controls” without incubation of the primary antibodies (Additional file 1: Figure S1C).

For double fluorescent immunostaining, the process of the antigen retrieval completely quenched the transgenically expressed GFP (Additional file 1: Figure S1A). After pre-incubation with blocking solution (5% normal donkey serum and 0.3% Triton X 100 in 0.1 M PBS), the sections were incubated with primary antibodies (i.e. α-syn antibody with the respective specific neuronal marker antibodies) and their paired double staining antibodies (Table 1) at room temperature for 1 h. The sections were mounted with an anti-fading medium for confocal microscopic analysis (Leica TCS SP8).

**Table 1:** Primary and secondary antibodies in double immunostainings

Double Immunostaining	1st antibodies	2nd antibodies
α- syn & PGP .5	Anti-α-syn: Santa Cruz Biotechnology, sc-12,767, mouse, dilution: 1/200	Cy3-donkey anti-mouse: Jackson ImmunoResearch, 715–165-150, dilution:1/200
	Anti-PGP 9.5: Abcam, ab72910, Chicken, dilution:1/800	Alexa 488 goat anti-chicken: Invitrogen, A11039, dilution: 1/1000
α- syn & α-syn (phosphoS129)	Anti-α-syn: Santa Cruz Biotechnology, sc-12,767, mouse, dilution: 1/200	Alexa 488-donkey anti-mouse: Jackson ImmunoResearch, 715–546-151, dilution:1/200
	Anti-α-syn (phosphoS129): Abcam, ab51253, rabbit, dilution:1/200	Cy3-donkey anti-rabbit: Jackson ImmunoResearch, 711–165-152, dilution:1/800
α- syn & calretinin	Anti-α-syn: Santa Cruz Biotechnology, sc-7011-r, rabbit, dilution: 1/200	Cy3-donkey anti-rabbit: Jackson ImmunoResearch, 711–165-152, dilution:1/800;
	Anti-calretinin: Santa Cruz Biotechnology, sc-11,644, goat, dilution:1/400	Alexa 488-donkey anti-goat: Jackson ImmunoResearch, 705–545-147, dilution:1/400
α- syn & nNOS	Anti-α-syn: Santa Cruz Biotechnology, sc-12,767, mouse, dilution: 1/200	Alexa 488-donkey anti-mouse: Jackson ImmunoResearch, 715–546-151, dilution:1/200
	Anti-neuronal nitric oxide synthase: Abcam, ab76067, rabbit, dilution:1/400	Cy3-donkey anti-rabbit: Jackson ImmunoResearch, 711–165-152, dilution:1/800
α- syn & VIP	Anti-α-syn: Santa Cruz Biotechnology, sc-12,767, mouse, dilution: 1/200	Alexa 488-donkey anti-mouse: Jackson ImmunoResearch, 715–546-151, dilution:1/200
	Anti-vasoactive intestinal polypeptide: Abcam, ab22736, rabbit, dilution:1/800	Cy3-donkey anti-rabbit: Jackson ImmunoResearch, 711–165-152, dilution:1/800

Quantification of the intensity of α-syn-GFP, calretinin, VIP and nNOS immunofluorescence was performed by measuring the mean fluorescence intensity in the positive area of the colonic cross-sections. The measurement of phospho-α-syn intensity was based on the gray value of immunohistochemistry staining in Auerbach’s plexus. The measurements of co-localization were processed using the Pearson’s Correction with the Fiji plugin Coloc 2. Two-way ANOVA tests were performed to statistically analyze the immunofluorescence intensity and co-localization, followed by Bonferroni adjustment post hoc tests with the software IBM SPSS Statistics 2.0.

## Results

### Age-dependent expression and accumulation of α-syn-GFP in the enteric neurons of the colon

**Fig. 1:**
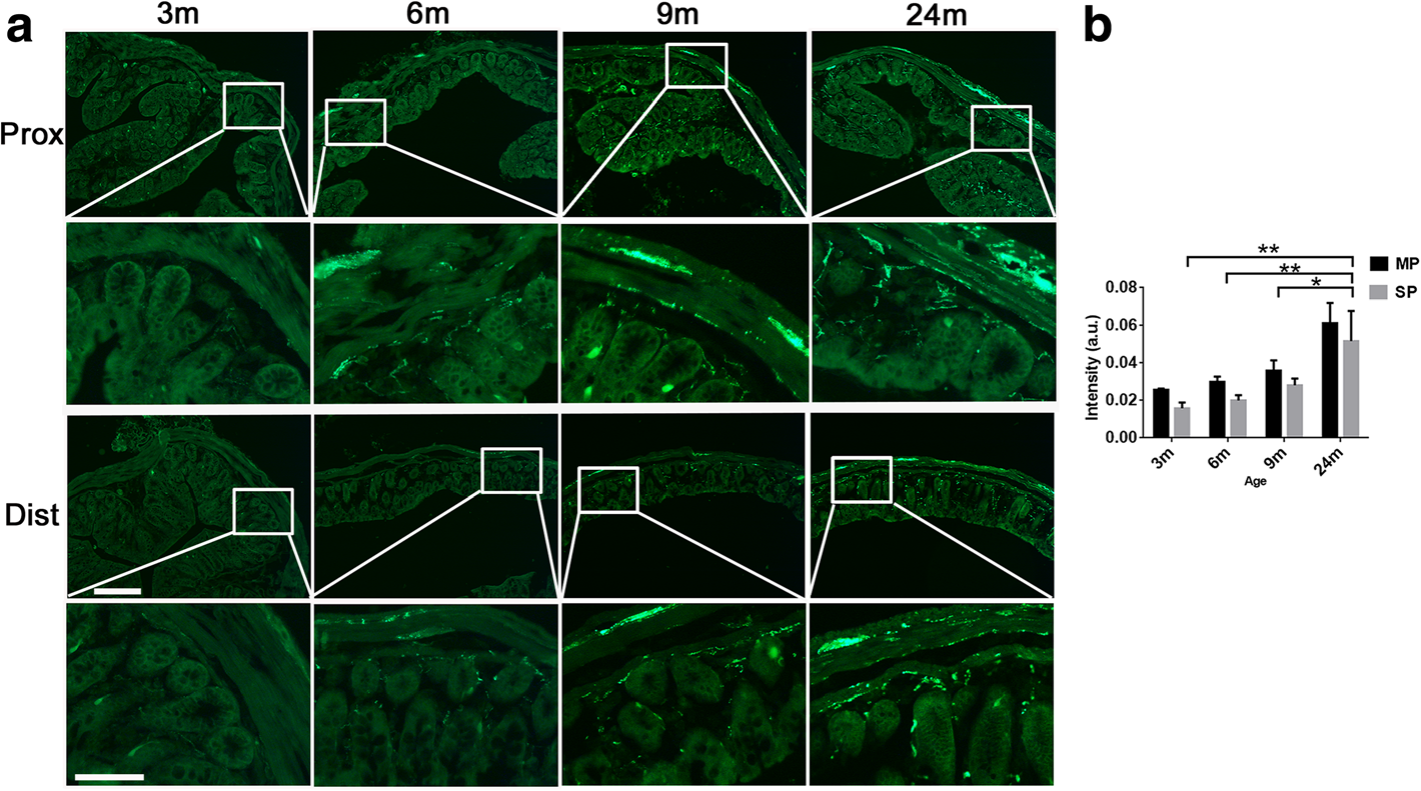
The presence of α-syn in enteric neurons of the colon with age. **a**. Representative images of α-syn-GFP transgene expression in the proximal (Upper panels) and distal (Lower panels) colon at 3, 6, 9 and 24 months of mice. **b**. The semi-quantitative analyses of α-syn fluorescence (GFP) intensity in the colon of transgenic mice at different ages [two-way ANOVA (F = 9.233, *p* = 0.001, in ages. F = 0.089, *p* = 0.98), followed by Bonferroni adjustment post hoc tests (^***^p<0.001, ^**^p<0.01, ^*^p<0.05)]. Error bars in SEM. Scale bar = 250 μm and 100 μm (zoomed up images) in **a**

We first analyzed the expression of α-syn-GFP in the colon in the proximal (close to the cecum) and distal (close to the rectum) regions. α-Syn-GFP was present in the nerve terminals in the mucosal layer and Auerbach’s and Meissner’s plexuses (Additional file 1: Figure S1B). The expression of α-syn-GFP was age-dependent; the transgene expression of α-syn-GFP in both the proximal and distal colon increased with age (Fig. 1). At 3 months of age, α-syn-GFP was weakly and sparsely present in the nerve fibers of the myenteric and the mucosal layers. From 6 months onwards, α-syn-GFP signals became more evident in the nerve fibers both in the myenteric and mucosal layers, and around the crypts of Lieberkühn (Fig. 1).

### Age-dependent phosphorylation of α-syn in the enteric neurons of the colon

**Fig. 2:**
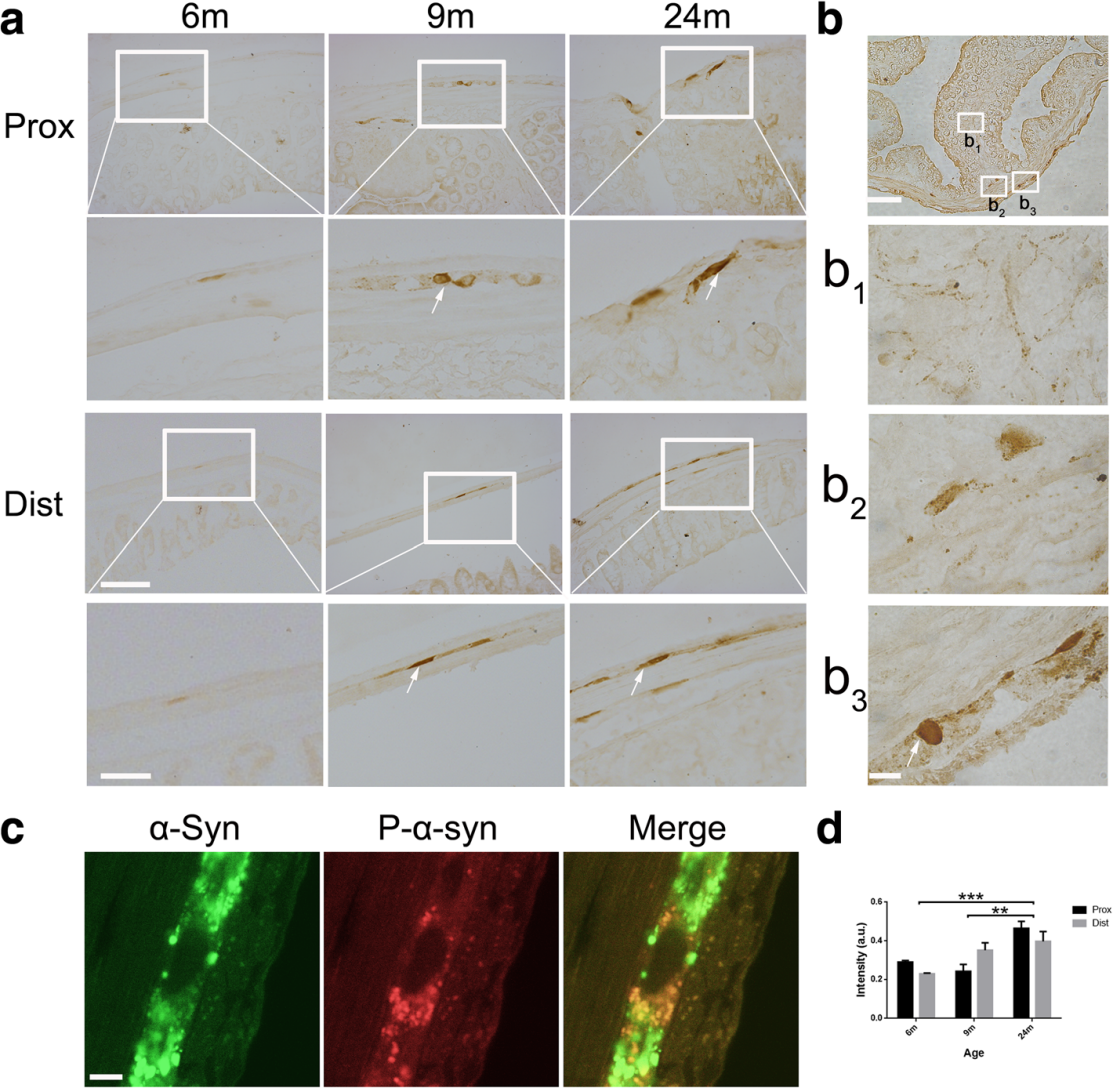
The accumulation of phospho-α-syn (pS129) in enteric neurons of the colon. **a**. The age-dependent phosphorylation of α-syn in the Auerbach’s plexus of the proximal (Upper panels) and distal (Lower panels) colon. **b**. Phospho-α-syn in the colon at the age of 18 months (b_1_, Mucosal layer. b_2_, Meissner’s plexus. b_3_, Auerbach’s plexus). **c**. Partial phosphorylation (phospho-α-syn (red)) in the Auerbach’s plexus, viewed as pan α-syn expression (green) (Pearson’s correction:0.388 ± 0.100). **d**. The semi-quantitative analyses of phospho-α-syn intensity (immunohistochemical staining) in the Auerbach’s plexus. Two-way ANOVA (F = 14.69, *p* = 0.0006, in ages. F = 0.5055, *p* = 0.8259), followed by Bonferroni adjustment post hoc tests (^**^p<0.01, ^**^p<0.001). Arrows show phospho-α-syn containing cell bodies of myenteric neurons . Scale bars = 250 μm and 100 μm (zoomed up images) in **a**; 500 μm and 50 μm in **b**; and 10 μm in **c**

As one of the most common post-translational modifications, phosphorylation of α-syn plays an important role in PD pathogenesis. We examined the status of α-syn phosphorylation (ser 129) in the enteric neurons of the colon of BAC-α-syn-GFP mice at different ages. At 6 months of age, phosphorylated α-syn was rarely detected in the enteric neurons in the proximal and distal colons of the mice (Fig. 2a). However, with age, phospho-α-syn became more abundant (Fig. 2d). α-Syn phosphorylation was more robust in the nerve fibers and terminals in Auerbach’s (myenteric) plexus, compared to Meissner’s plexus. Interestingly, phospho-α-syn containing cell bodies of myenteric neurons were observed (Fig. 2a and b, arrows). Partial co-localization of pan α-syn and phospho-α-syn was detected in Auerbach’s plexus (Fig. 2c).

### α-Syn(-GFP) in subsets of enteric neurons in the colon

**Fig. 3:**
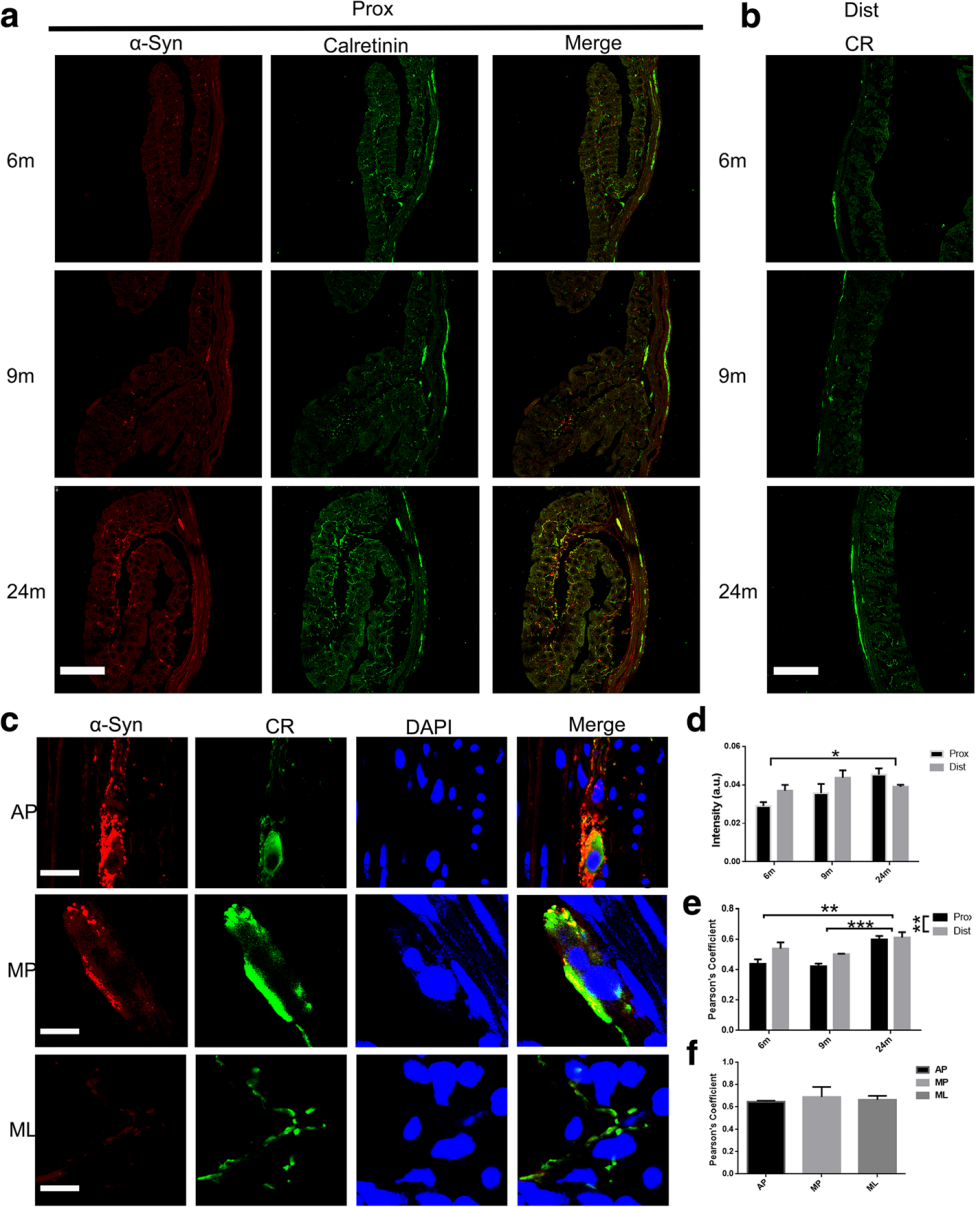
Co-localization between calretinin and α-syn in enteric neurons in the colon. **a** α-syn and calretinin expression in the enteric neurons increased with age and highly co-localized to each other at 6, 9, and 24 months in the proximal colon. **b** Expression of calretinin in the distal colon also increased with age. **c** Co-localization between calretinin (CR) and α-syn in the enteric nerve terminals and neuronal cell bodies in the mucosal layers (ML) and Auerbach’s (AP) and Meissner’s plexuses (MP) at 24 months. **d** Semi-quantitative analyses of calretinin in the colon of BAC α-syn-GFP mice. Two-way ANOVA (F = 6.277, *p* = 0.014, in ages. F = 0.420, *p* = 0.529), followed by Bonferroni adjustment post hoc tests (^*^p<0.05). **e** Quantitative of co-localization between calretinin and α-syn in the colon in BAC α-syn-GFP mice. Two-way ANOVA (F = 1.442, *p* = 0.275, in ages. F = 10.840, *p* = 0.006). **f** Quantitative of co-localization between calretinin and α-syn in the mucosal layers (ML) and Auerbach’s (AP) and Meissner’s plexuses (MP) 24 months. Scale bar = 250 μm in **a** and **b**; 20 μm in **c** (MP, ML) and 40 μm in **c** (AP)

**Fig. 4:**
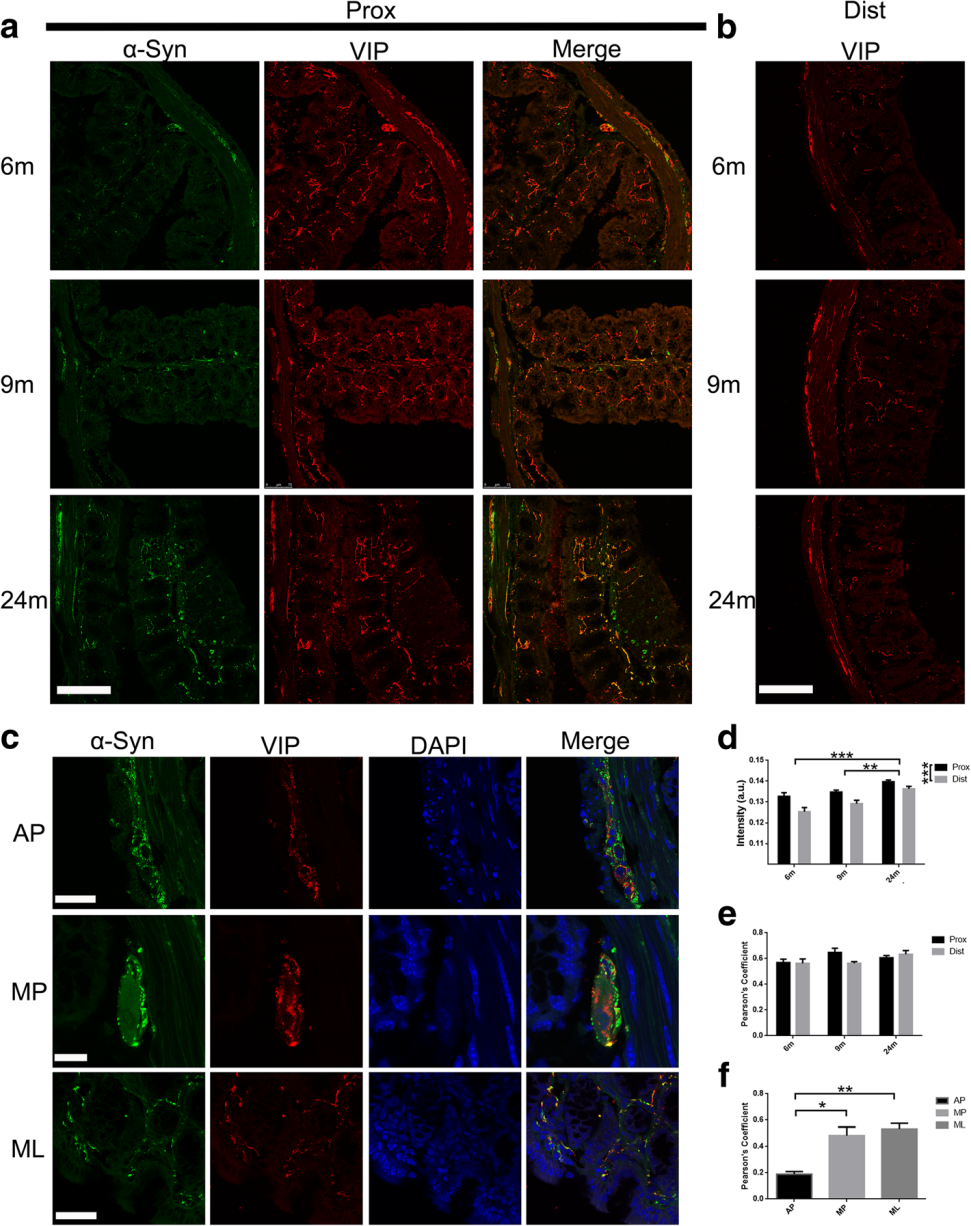
Co-localization between VIP and α-syn in enteric neurons of the colon. **a** and **b**, Age-dependent increase of VIP expression was observed in the enteric neurons of the proximal colon at 6, 9, and 24 months, partial co-localization of VIP and α-syn was seen at 24 months old of age. **c** Co-localization of VIP and α-syn, and the nucleus (DAPI) at 24 months in mucosal layers (MP) and Auerbach’s (AP) and Meissner’s (MP) plexuses. **d** Semi-quantitative analyses of VIP in the colon of BAC α-syn-GFP mice. Two-way ANOVA [F = 18.999, *p* = 0.0002, in ages. F = 19.950, *p* = 0.0008(^***^p), followed by Bonferroni adjustment post hoc tests (^***^p<0.001, ^**^P<0.01). **e** Quantitative of co-localization between VIP and α-syn in the colon in BAC α-syn-GFP mice. Two-way ANOVA (F = 2.150, *p* = 0.159, in ages. F = 0.950, *p* = 0.349)]. **f** Quantitative of co-localization between VIP and α-syn at 24 months in mucosal layers (MP) and Auerbach’s (AP) and Meissner’s (MP) plexuses. Student *t* test (^*^p<0.05, ^**^p<0.01). Scale bar = 150 μm in **a** and **b**; 50 μm in **c** (AP, ML) and 15 μm in **c** (MP)

**Fig. 5:**
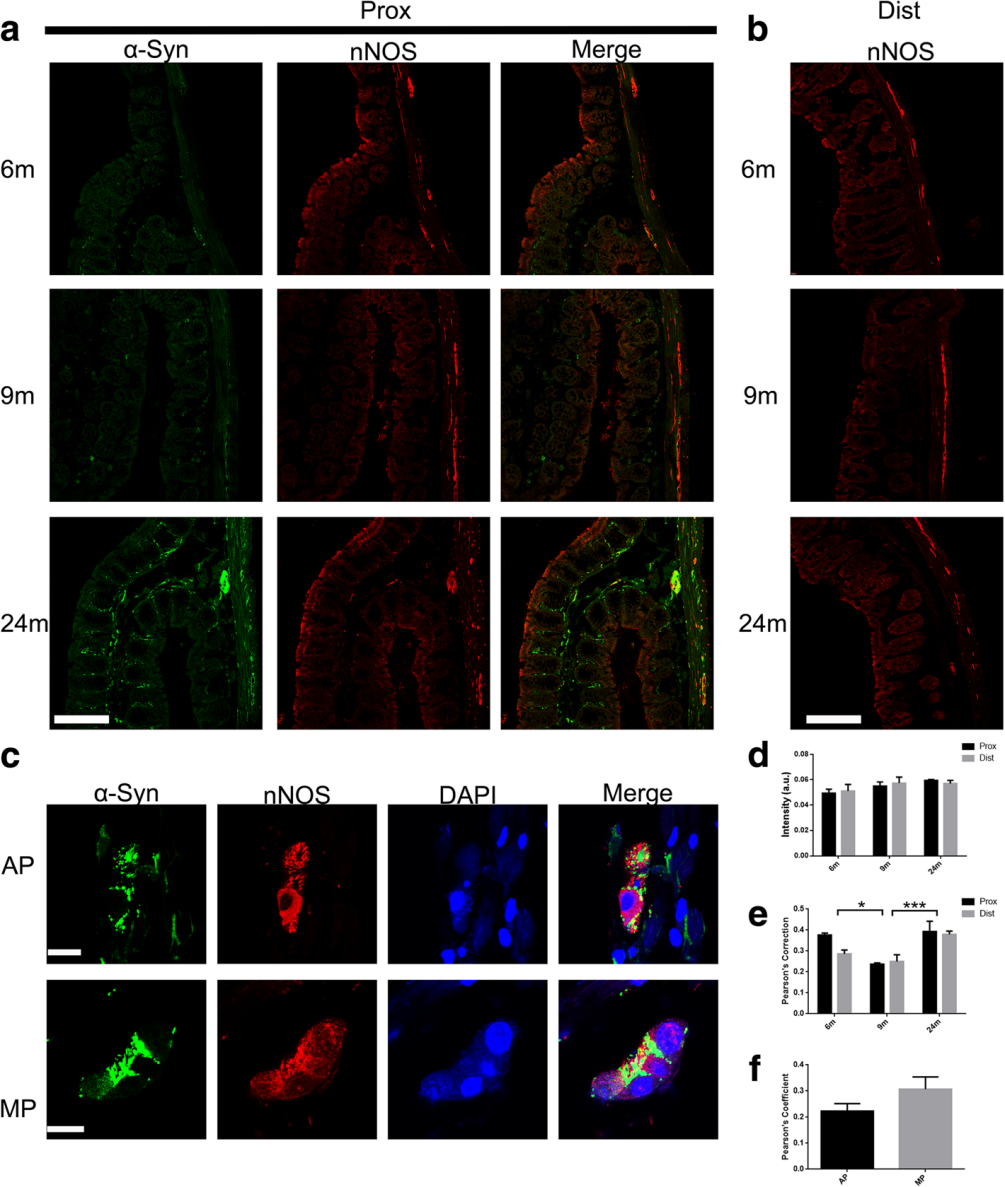
Expression of nNOS in enteric neurons of the colon of transgenic mice and co-localization of nNOS and α-syn. **a** nNOS expression is predominant in the Auerbach’s plexus without clear age-related changes. **b** Expression of nNOS in distal colon at 6, 9, and 24 months. **c** Co-localization of nNOS and α-syn, and the nucleus (DAPI) at 24 months in Auerbach’s (AP) and Meissner’s (MP) plexuses. **d** Semi-quantitative analyses of nNOS in the colon of BAC mice. Two-way ANOVA (F = 2.723, *p* = 0.106, in ages. F = 0.031, *p* = 0.862). **e** Quantitative of co-localization between nNOS and α-syn in the colon in BAC mice. Two-way ANOVA (F = 15.262, *p* = 0.001, in ages. F = 1.970, *p* = 0.186), followed by Bonferroni adjustment post hoc tests (^*^p<0.05,^***^P<0.001). **f** Quantitative of co-localization between nNOS and α-syn at 24 months in Auerbach’s (AP) and Meissner’s (MP) plexuses. Scale bar = 150 μm in **a** and **b**; and 15 μm in **c**

Different subtypes of neurons secrete different transmitters and regulate colonic function. Calretinin plays an important role in PD: striatal calretinin neurons are sensitive to dopamine depletion and calretinin in dopaminergic neurons may have a protective function for their survival [14–16]. The number of nNOS-expressing interneurons is up-regulated in PD and nNOS is thus implicated in the disease pathogenesis [17]. On the other hand, the treatment with VIP can reverse the motor deficits in the 6-hydroxydopamine (6-OHDA) lesioned model of PD [18]. Thus, we analyzed these three subtypes of neurons in the colon by double immunofluorescence labeling with the α-syn antibody and the respective neuronal subtype-specific marker. We observed that calretinin was widely expressed in both Auerbach’s and Meissner’s plexuses and nerve fibers in different colonic layers. The expression of calretinin increased with age (Fig. 3a, b and d). Calretinin and α-syn (-GFP) highly co-localized in the two plexuses and in the nerve fibers of all the layers (mucosal, submucosal and myenteric layers) (Fig. 3c and f). the extent of co-localization increased with age (Fig. 3e). Similarly, VIP was broadly expressed in both Auerbach’s and Meissner’s plexuses and nerve fibers in different colonic layers and it was increased in both the proximal and distal colon with age (Fig. 4a, b and d). VIP was highly co-localized with α-syn (-GFP) in the mucosal layer fibers and Meissner’s plexus, but, to a lesser extent, in Auerbach’s plexus (Fig. 4c and f). nNOS, on the other hand, was expressed in Auerbach’s and Meissner’s plexuses and in nerve fibers of the muscular layer (Fig. 5a and b), but not present in the nerve fibers in submucosal and mucosal layers. The expression of nNOS in the proximal and distal colon showed no change with age (Fig. 5a, b and d), which is not consistent with the changes in the small intestine we previously reported [13]. Apart from this, nNOS was poorly co-localized with α-syn (-GFP) in both Auerbach’s and Meissner’s plexuses (Fig. 5c and f).

## Discussion

A large body of clinical reports have suggested that gastrointestinal dysfunction, such as constipation, is a common non-motor symptom in PD, starting from the prodromal stage and lasting throughout the course of the disease [19, 20]. The links between the gut and the brain are manifold and both direct and indirect in PD development. Braak and colleagues have suggested that α-syn pathology could be initiated in the gut and then propagate to the brain. In support of this hypothesis, our lab has previously reported the direct spread of pathological α-syn from the small intestine to the lower brain stem via the vagal nerve. Additionally, there have been studies suggesting a connection of PD progress and gut inflammation. Bialecka et al. reported that the frequency of CARD15/NOD2 gene variants, associated with Crohn’s disease, may be a risk factor for sporadic PD development [19]. In 2013, David et al. also found pro-inflammatory cytokines, glial fibrillary acidic protein and Sox-10 to be elevated in the ascending colon of PD patients [20]. Other studies have provided evidence of intestinal inflammation in PD patients through fecal examinations [21, 22]. Moreover, intestinal microbiota in PD patients were found to differ from those in healthy controls: a decrease of anti-inflammatory, butyrate-producing bacteria and an increase in pro-inflammatory bacteria were observed in PD patient stool samples compared to those of healthy controls [20, 21]. In experimental models, microbiota has similarly been shown to regulate movement deficits providing further support to the link between intestinal inflammation and PD pathology [22].

It is of importance to understand the involvement of the gastrointestinal system in initiation and development of PD pathology. Our BAC-α-syn-GFP mice possess both the symptomatic and pathological features of early PD. α-Syn-GFP accumulation and phosphorylation were found increased with age in the colon of the transgenic mice, which corresponds to our observations of an increased gut transit time (Additional file 2: Figure S2), implying a sign of constipation symptoms in mice. Regarding motor symptoms, in our previous study, the BAC-α-syn-GFP mice displayed progressive behavioral impairment in open field and rotarod tests at 7 and 12 months of age [12]. Interestingly, in the colon of the mice, phosphorylated α-syn was detected prior to the onset of motor behavioral deficits, indicating that the pathological alterations in the gut appear before the motor deficits even occur. The differential co-localization between α-syn (-GFP) with calretinin, VIP and nNOS suggests that different subtypes of enteric neurons may possess a distinct role in gastrointestinal dysfunction in PD.

## Conclusions

Using our BAC-α-syn-GFP mouse model, we have demonstrated an age-dependent accumulation, phosphorylation and aggregation of α-syn in the enteric neurons of the colon. Our model presents both the symptomatic and pathological features of early PD, expanding from the gut to the brain. The data indicate that this mouse model possesses the uniqueness and potential for studying PD gastrointestinal dysfunction.

## Additional files

Additional file 1: **Figure S1.** A. The quenched α-syn-GFP signal, double immunofluorescence images labeled for PGP 9.5 and α-syn, and the phospho-α-syn immunohistochemical staining in the colon. (TIF 63429 kb)

Additional file 2: **Figure S2.** The gut transit time in the BAC and wild type male mice with age. (TIF26 kb)

## Abbreviations

6-OHDA: 6-hydroxydopamine
BAC: Bacterial artificial chromosome
C57BL/6: c57 black 6
GFP: Green fluorescent protein
LB: Lewy bodies
LN: Lewy neurites
nNOS: neuronal nitric oxide synthase
PBS: Phosphate buffered saline
PD: Parkinson’s disease
PFA: Paraformaldehyde
VIP: Vasoactive intestinal peptide
α-syn: α-synuclein
