# Supplementary figures and images for: Age-dependent alpha-synuclein accumulation and aggregation in the colon of a transgenic mouse model of Parkinson’s disease
Source: Transl Neurodegener. 2018 Jun 30;7:13. doi: 10.1186/s40035-018-0118-8 (PMC6026335; doi:10.1186/s40035-018-0118-8)

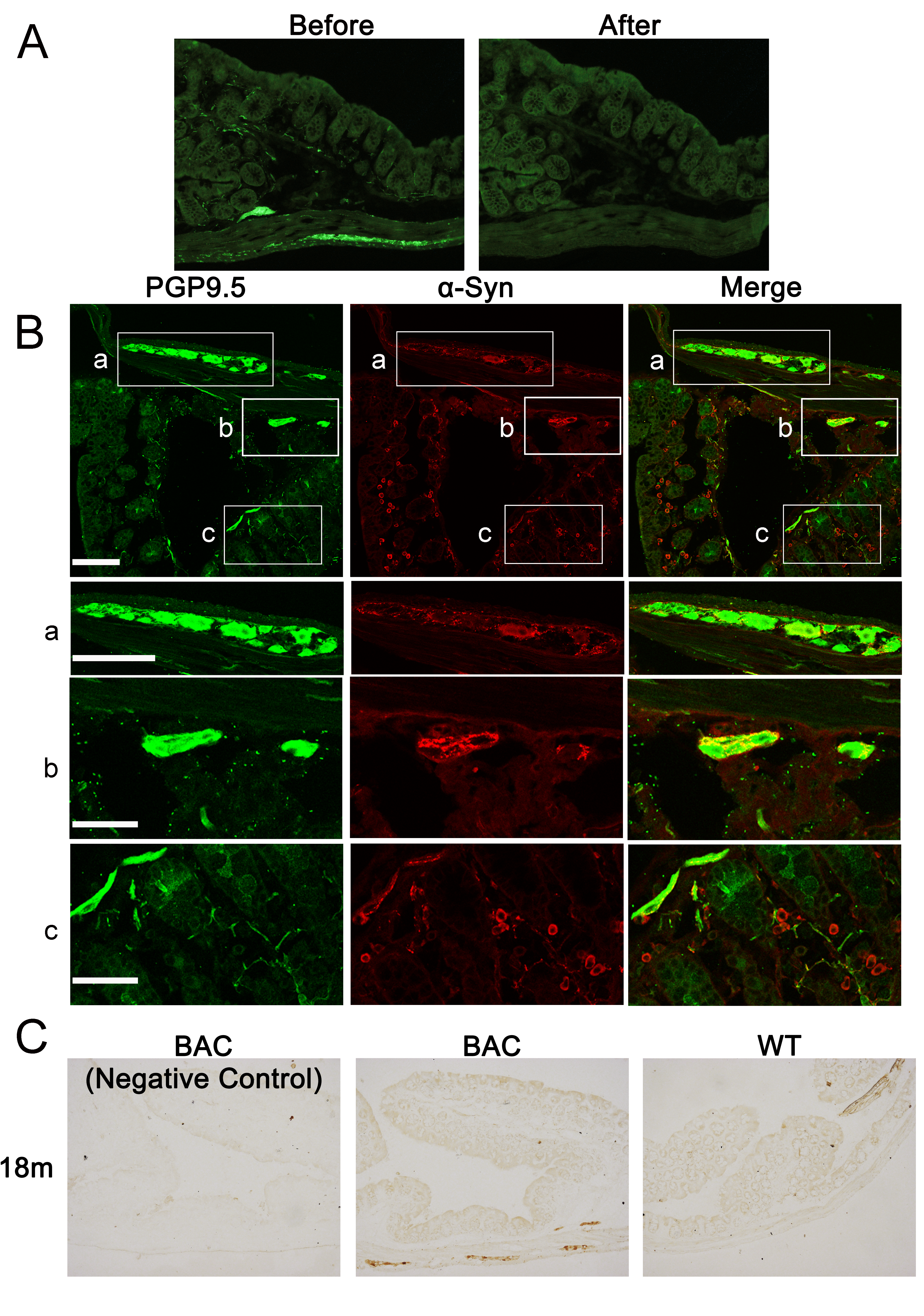

Supplement: Supplementary file 1 — Figure S1. A. The quenched α-syn-GFP signal, double immunofluorescence images labeled for PGP 9.5 and α-syn, and the phospho-α-syn immunohistochemical staining in the colon. (TIF 63429 kb) [file 40035_2018_118_MOESM1_ESM.tif]
